# Guillain–Barré Syndrome in war-torn regions: the forgotten neurological emergency in humanitarian crises

**DOI:** 10.1097/MS9.0000000000004125

**Published:** 2025-10-21

**Authors:** Zahabia Adnan, Amna Kashif, Ahmed Asad Raza, Abedin Samadi

**Affiliations:** a Department of Medicine, Jinnah Sindh Medical University, Karachi, Pakistan; b Department of Medicine, Kabul University of Medical Sciences, Kabul, Afghanistan

**Keywords:** conflict zones, global health, Guillain–Barré syndrome, humanitarian crises, neurological emergency

## Abstract

Guillain-Barré Syndrome (GBS) is a life-threatening neurological emergency and the leading cause of acute flaccid paralysis in the post-polio era. While extensively studied in stable healthcare systems, its impact in conflict-affected regions remains underrecognized. This paper highlights GBS as a neglected but significant health crisis in war-torn settings, where collapsing sanitation systems, overcrowded shelters, and widespread infections act as key triggers. Data from recent humanitarian emergencies, including Gaza, reveal rising incidence and mortality due to inadequate surveillance, scarce diagnostic capacity, and lack of access to intravenous immunoglobulin or plasmapheresis. Unlike high-income countries, where timely ICU care keeps mortality under 5%, patients in low-resource conflict zones often experience prolonged disability or death. Addressing this disparity requires practical, low-cost interventions such as early recognition training for frontline workers, provision of essential supportive care, and international advocacy for humanitarian supply corridors. Remote digital consultations and safe patient evacuation can further enhance outcomes. Incorporating GBS into humanitarian response frameworks is an urgent necessity to reduce preventable neurological deaths. By bringing attention to this overlooked emergency, the manuscript underscores the moral and medical imperative of ensuring equitable neurological care during crises.

In conflict zones, global attention is often consumed by the visible toll of war with bullets, bombings, famine, and trauma overshadowing silent emergencies, particularly neurological ones, that often go undetected. A sudden onset and potentially fatal autoimmune disorder that attacks the peripheral nerves and has emerged as one of the major overlooked crises. Guillain-Barré Syndrome (GBS) is now the leading cause of acute flaccid paralysis in the post-polio era while remaining almost entirely absent from humanitarian response interventions^[1]^. The situation is no longer theoretical. In Gaza, for example, the World Health Organization has documented a sharp rise in GBS cases to 94 patients and 10 deaths since June 2025, owing to collapsing sanitation systems, rampant infections, and depleted medical supplies^[2]^. This exacerbation highlights both the urgency and the neglect this problem continues to face.

Even in the absence of war, GBS is a formidable neurological emergency. Global incidence is estimated at 1–2 cases per 100 000 people per year, and mortality reaches up to 7–8% despite access to critical care^[3]^. This exemplifies how quickly GBS can overwhelm a health system even in relatively stable environments. A significantly worse picture is seen in lower-middle-income countries as a consequence of delayed diagnosis, high treatment cost, as well as a limited access to essential interventions, putting patients at risk of prolonged disability or death. A case series from Syria has documented GBS linked to COVID-19, highlighting how fragile systems struggle to respond to such a devastating disease^[4]^. But Gaza today represents a new inflection point as GBS is emerging not as a complication of a pandemic but as a neurological emergency as a direct consequence of war, exemplifying the distinct double burden of inequity faced when already fragile health systems are further dismantled by conflict.

One of the major leading causes of GBS in conflict-zone areas is the intake of contaminated drinking water and poor sewage disposal, which leads to the prevalence of gastrointestinal infections, particularly *Campylobacter jejuni*^[5]^. This is due to the collapse of Water, Sanitation, and Hygiene systems in war settings. Similarly, respiratory infections and micronutrient deficiencies are also considered key triggers of GBS, which is common because of the destruction of health infrastructure and overcrowded shelters. Such disorders are often under-reported because of weak surveillance systems that lead humanitarian agencies to perceive the condition as a low-priority issue, giving rise to clusters of cases, as seen in Gaza and other war-affected areas. Treatment of this neurological emergency is highly resource-intensive, placing a significant burden on the healthcare system. Beyond supportive care, intravenous immunoglobulin (IVIG) and plasmapheresis are two main disease-modifying therapies that are expensive, require specialized equipment, cold-chain storage, and are rarely available in conflict conditions. However, with quick access to ICU support and timely immunotherapy, GBS mortality rates are normally below 5% in well-resourced healthcare settings^[6]^.

Under siege conditions, where blockade and bombardments are common, it is nearly impossible to follow an ideal humanitarian framework by giving ICU-level care. A realistic call to action in places such as Gaza is to implement low-cost, practical, and scalable interventions that can instantly save human lives. The most genuine approach is to prioritize early recognition by training frontline health workers about simple clinical symptoms such as loss of reflexes, recent infections, or progressive symmetrical weakness. This can be associated with basic palliative care, including oxygen concentrators, ventilatory support, and rehabilitation facilities for the survival of patients. Such interventions are considered less expensive than immunotherapy, but can still play a critical role in reducing both complications and deaths^[7]^. Among possible external responses, the most realistic action is advocacy of humanitarian supply corridors, including the supply of IVIGs and plasma exchange filters alongside trauma kits and antibiotics for neurological emergencies like GBS. Moreover, severely affected patients should be safely evacuated to nearby referral centres due to the breakdown of local capacity. More funds should be collected across the globe to make this happen. Additionally, sharing data digitally can provide remote support by international neurologists to improve clinical judgment on a patient’s condition and management.

GBS in conflict zones represents a forgotten neurological emergency that slips through the cracks of humanitarian response. Despite being treatable and preventable, it is present in its worst outcomes in areas like Gaza. What is urgently needed is the incorporation of these conditions into emergency response strategies that could save people’s lives from harsh outcomes.

To ensure methodological transparency and ethical compliance in manuscript preparation, we adhered to the recently developed TITAN (Transparency in the Reporting of Artificial Intelligence) 2025 guidelines, which outline standards for responsible AI use in biomedical writing and publishing^[8]^.

## Data Availability

No datasets were generated or analyzed during the current study.
